# Analysis of *ProP1* Gene in a Cohort of Tunisian Patients with Congenital Combined Pituitary Hormone Deficiency

**DOI:** 10.3390/jcm11247525

**Published:** 2022-12-19

**Authors:** Mariam Moalla, Mouna Mnif-Feki, Wajdi Safi, Nadia Charfi, Nabila Mejdoub-Rekik, Mohamed Abid, Faten Hadj Kacem, Hassen Hadj Kacem

**Affiliations:** 1 Laboratory of Molecular and Cellular Screening Processes, Center of Biotechnology of Sfax, Sfax 3018, Tunisia; 2 Laboratory of Human Molecular Genetics, Faculty of Medicine of Sfax, University of Sfax, Sfax 3029, Tunisia; 3 Endocrinology Department, Hedi Chaker Hospital, Sfax 3029, Tunisia; 4 Department of Applied Biology, College of Sciences, University of Sharjah, Sharjah P.O. Box 27272, United Arab Emirates

**Keywords:** non-syndromic combined pituitary hormone deficiency, *ProP1* gene, Sanger sequencing, p.(Gln114Ter), p.Arg73Cys

## Abstract

Background: Non-syndromic combined pituitary hormone deficiency (CPHD) occurs due to defects in transcription factors that govern early pituitary development and the specification of hormone-producing cells. The most common mutations are in the Prophet of Pit-1 (*ProP1*) gene. This work aims to (1) report findings of genetic analyses of Tunisian patients with non-syndromic CPHD and (2) describe their phenotype patterns and their evolution through life. Methods: Fifteen patients from twelve unrelated families with variable clinical phenotypes were included after excluding autoimmune and acquired forms of non-syndromic CPHD. Detailed pedigree charts and auxological, hormonal, radiological, and therapeutic details were recorded. Sanger sequencing was performed, and sequences were analyzed with a specific focus on coding and splice site regions of the *ProP1* gene. Retained variants were classified using several in silico pathogenicity prediction tools and the VarSome platform. Results: We identified the common p.Arg73Cys mutation in seven patients from four unrelated pedigrees. We found a novel homozygous mutation (c.340C>T) in one sporadic case. This mutation generates a truncated ProP1 protein, predicted to be non-functional, lacking the last 112 codons (p.(Gln114Ter)). We confirmed by polymerase chain reaction (PCR) the absence of large exon deletions or insertions in the remaining sporadic patients (7/8). Conclusions: We report two mutations {one newly identified [p.(Gln114Ter)] and one previously reported (p.Arg73Cys)} in five unrelated Tunisian families with non-syndromic CPHD. This work is of clinical importance as it reports the high frequency of the p.Arg73Cys mutation in Tunisian CPHD families. Our study also illuminated the involvement of novel gene(s) in the emergence of non-syndromic CPHD.

## 1. Introduction

Combined pituitary hormone deficiency (CPHD) is a disease characterized by the impaired production of at least two anterior pituitary hormones and can vary in severity and age at presentation [1].

Altered expression, in time and space, of about 30 genes encoding transcription factors and signaling molecules leads to CPHD [2]. To date, a few mutations in these genes have been reported with various inheritance patterns [1,3]. As a general rule, mutations in genes expressed early in the pituitary developmental process (such as *HESX1*, *SOX2*, *SOX3*, *LHX3*, *LHX4* genes) lead to various combinations of hormonal deficiencies, sometimes associated with extra-pituitary birth defects mostly affecting the brain, the eye or craniofacial structures [2]. In contrast, mutations in genes acting later in the process (such as *ProP1* and *POU1F1* genes) result in variable endocrine phenotypes of CPHD without any extra-pituitary abnormalities [2]. 

Prophet of Pit-1 (*ProP1*) is the most frequently mutated gene known to cause autosomal recessive non-syndromic CPHD in both familial and sporadic cases [1]. This gene is located on the long arm of chromosome 5 (5q35.3), consists of three highly conserved exons, and encodes a transcription factor of 226 amino acids [4,5]. To date, at least 30 *ProP1* point mutations (missense, splicing variants, nonsense, mutations affecting the initiation codon) and frameshift small insertions and deletions have been reported [6]. Most of these are located within the second exon with a remarkably uneven regional/ethnic distribution of mutations (0.8–65%) [6,7]. Homozygous deletions of the entire gene or particular exons were also reported in nine families of different ethnic origins [8,9,10,11,12,13,14]. In humans, recessive *ProP1* mutations cause growth hormone (GH), thyroid-stimulating hormone (TSH), luteinizing hormone (LH), follicle-stimulating hormone (FSH), and prolactin (PRL) deficiencies [1]. Adrenocorticotropic hormone (ACTH) deficiency can evolve later in some *ProP1* patients and, therefore, ongoing monitoring is needed [1,15,16]. The onset and severity of anterior pituitary hormone deficiencies, as well as changes in pituitary morphology, are highly variable in *ProP1* patients [1].

Systemic *ProP1* testing provides important diagnostic, prognostic and therapeutic implications for mutation-carrier patients and supports prenatal diagnosis for affected and unaffected carriers’ parents. The clinical utility of *ProP1* testing emerges over time through the collection of detailed phenotypic and hormonal data from *ProP1* mutation-negative patients presented with all anterior pituitary hormone deficiencies. This approach may facilitate novel disease-gene discovery and expand knowledge regarding developmental and functional pathways of the hypothalamic–pituitary axes.

We undertook this study to uncover *ProP1* gene defects in a cohort of Tunisian patients with familial and sporadic non-syndromic CPHD.

## 2. Materials and Methods

### 2.1. Subjects

**Figure 1:**
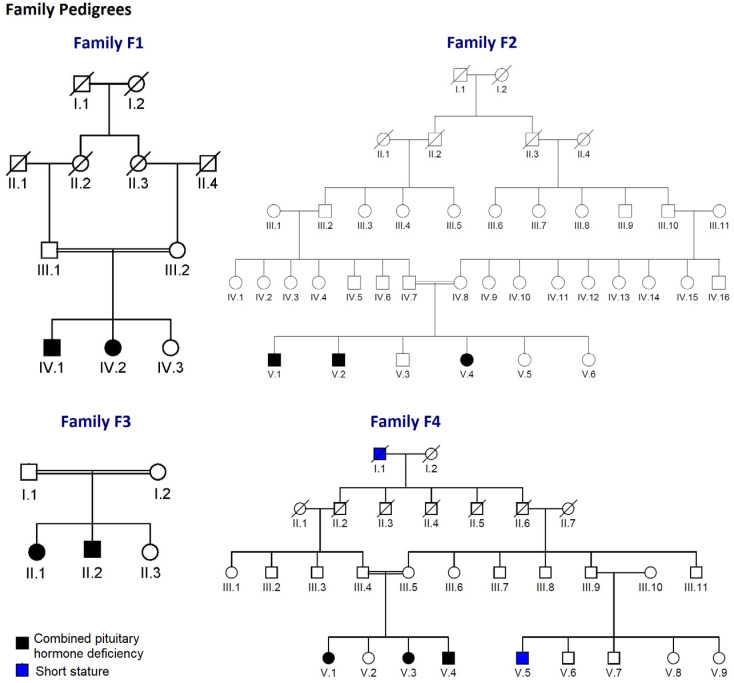
Pedigree of the studied families. The generations within the family are indicated by roman numerals. Squares and circles represent male and female, respectively. Normal individuals are shown as clear symbol, whereas the affected individuals are shown as filled symbol. Patients F3-II.2, F4-V.1 and F4-V.3 consult in private clinics and refuse hormonal assessment and genetic testing.

The present study included 15 patients from 12 unrelated families from different regions of Tunisia diagnosed with non-syndromic CPHD. Among these patients were eight sporadic patients and four familial index cases (a total of seven familial patients with available DNA). Consanguineous marriages were observed in four of the recruited families (Figure 1). In addition, a control group composed of 211 Tunisian subjects was used.

All participants were informed about the purpose of the study and their written consent was obtained prior to embarking on this genetic study. Parental consent was required for patients under 18 years of age. The study protocol was conducted following the guidelines of the Helsinki Declaration, and the Regional Committee of the Protection of Persons of Sfax, Tunisia, approved the use of anonymized DNA samples (CPP SUD N°28/2019).

Clinical, biochemical, and neuro-radiological data were collected from each patient by physicians at the Department of Endocrinology of the Hedi Chaker University Hospital (Sfax, Tunisia). Detailed family histories were also recorded for all patients.

The clinical data included a perinatal history of patients (week of gestation, type of delivery, birth length/weight, hypoglycemic episodes, cryptorchidism/micropenis), auxological data collected upon admission and during the follow-up (weight, height, body mass index (BMI), palpation of thyroid, sexual maturity rating according to Tanner staging, hormonal replacement therapy), parents’ heights and puberty dates, and family history of endocrine diseases.

Patients included in the study had at least one anterior pituitary hormone deficiency at diagnosis (Table 1). Basal serum levels of TSH (normal range: 0.27–4.2 μU/mL), free thyroxine 4 (FT4; 12–22 pmol/L), FSH [males: 1.5–12.4 mU/mL; women (follicular phase): 3.5–12.5 mU/mL], LH [males: 1.7–8.6 mU/mL; women (follicular phase): 2.4–12.6 mU/mL], testosterone [males: 2.49–8.36 ng/mL; females: 0.084–0.481 ng/mL], estradiol [males 25.8–60.7 pg/mL; females (follicular phase): 12.4–233 pg/mL], PRL [males: 3.46–19.4 ng/mL; females (non-pregnant): 4.79–23.3 ng/mL], cortisol [60–184 ng/mL (6 h–10 h)] and basal ACTH (7.2–63.3 pg/mL) were measured at 08:00 a.m. Thyreotroph deficiency was confirmed when a low plasma FT4 level was associated with a low or inappropriately normal TSH level [17]. Gonadotropin deficiency was diagnosed based on clinical signs (cryptorchidism, micropenis, labial hypoplasia in the postpartum period, and/or absence of pubertal development at 14 years in boys and 13 years in girls) and hormonal findings [low basal levels of gonadotropins and sex steroids with no/partial increase in LH and FSH levels following infusion of gonadotropin-releasing hormone (GnRH; 100 μg)] [17]. Corticotroph deficiency was confirmed when low/normal ACTH levels were measured in the presence of low basal cortisol levels (less than 40 ng/mL in neonates and 80 ng/mL in older children) with no increase during the insulin-induced hypoglycemia (IIH) test (Actrapid insulin, 0.1 IU/kg, at 30 min post-stimulation corresponding to an adequate venous blood glucose nadir) and/or the stimulatory Synacthen test (1 μg, at 30 and 60 min) [17]. GH secretion capacity was assessed by different pharmacological stimulation tests [IIH or avlocardyl (0.75 mg/Kg)-glucagon (1 mg) tests]: Complete and partial GH deficiency was confirmed when the GH peak was below 5 ng/mL and between 5–10 ng/mL, respectively [17]. Hormone levels were measured using the electrochemiluminescence immunoassay (ECLIA) method on a Roche Cobas 6000 Analyzer according to the manufacturer’s instructions for calibration and sample processing.

Skeletal maturation was determined using the Greulich–Pyle method and bone age delay was calculated as the difference between skeletal and chronological age. Brain and pituitary magnetic resonance imaging (MRI) was carried out in all patients and interpreted by at least two trained neuroradiologists.

Patients with acquired causes of hypopituitarism secondary to neurological insult, autoimmune disease, infections, and infiltrative disorders (hypothalamic or pituitary tumors) were excluded from the study.

### 2.2. Genetic Examinations

Genomic DNA was extracted from peripheral blood cells using the classical phenol-chloroform extraction protocol [18]. DNA was quantified spectrophotometrically using a Qubit^®^ 2.0 Fluorometer (Invitrogen, CA) as per the manufacturer’s instructions.

The coding gene sequences, together with ~50 nucleotides from neighboring intronic and regulatory regions, were amplified by polymerase chain reaction (PCR) using a set of specific primers available on request. Primers were designed using the Refseq gene sequence (NM_006261; NP_006252) and Primer3 software V4.1.0 (Koressaar; Tartu, Estonia) [19]. PCR products were purified using the PureLink Quick Gel Extraction Kit (Thermo Fisher Scientific) and subsequently bi-directionally sequenced on an ABI 3100-4 Automated Genetic Analyzer (Applied Biosystems Inc., Foster City, CA, USA) as per the manufacturer’s instructions. *ProP1* sequence data were examined for mutations using the Bioedit software V7.1.3 (Tom Hall; Carlsbad, CA, USA) and the reference gene sequence.

Each identified variant was checked in online population allele frequency databases (Single Nucleotide Polymorphism Database (dbSNP) [20], 1000 genomes browser database [21], and GnomAD database V2.1.1 [22,23]). Subsequently, mutant variants were interpreted by in silico prediction tools for amino acid changes (SIFT [24], Polyphen-2 [25], PROVEAN [26] and Ensembl Variant Effect Predictor [27]) and splice site modification (Human Splice Finder V3.1 [28]). The rare new pathogenic variants identified were classified according to the American College of Medical Genetics and Genomics (ACMG) criteria using the VarSome v10.2 platform [29].

Retained variants were tested for co-segregation in all available healthy relatives and controls by Sanger sequencing.

## 3. Results

### 3.1. Cohort Description

Clinical features and hormonal evaluation of all patients regarding their genetic status are presented in Table 1. Patients’ first examination was performed during childhood (67%), adolescence (27%), and adulthood (6%). At diagnosis, all patients were short compared to the target height. A delay in bone age was observed in all patients (at least two standard deviations) compared to chronological age.

All patients lacked a GH response to provocative tests, except for the S6 patient, who showed a partial response (Table 1). They displayed absent pubertal development, except for patients S5 and S10 who were diagnosed during the prepubertal period. Hormonal assessments revealed very low basal levels of sex steroids and gonadotropins (Table 1). Seventy-four percent (11/15) of patients had central hypothyroidism at their last follow-up (Table 1). Basal ACTH levels were normal in all patients (Table 1). ACTH stimulation tests performed upon admission yielded variable responses ranging from a normal increase in cortisol levels (only for cases F3-II.1 and S3) to a blunted response (for the remaining cases). Repeated ACTH stimulation tests at the follow-up in patients F3-II.1 and S3 showed decreased peak cortisol levels of 156 and 154 ng/mL, respectively, indicating a progressive course of adrenal insufficiency (Table 1). Our study reports that 53% (8/15) of cases had normal serum prolactin levels during the last follow-up (Table 1). Regular hormone therapy was started upon diagnosis of the deficiency and consisted of the administration of GH, L-thyroxine, hydrocortisone, and sex steroids. All patients responded well to the appropriate hormone replacement.

Pituitary morphology abnormalities were found in 87% (13/15) of cases (Table 1). Among our cohort of 15 patients, MRI showed an empty sella turcica in one patient (F1-IV.2) and pituitary hypoplasia in two patients (F2-V.4 and S11). Four patients had anterior pituitary hypoplasia with normal neurohypophysis (F2-V.1, F3-II.1, S6) and a small-sized sella turcica (F2-V.2). Pituitary stalk interruption syndrome was observed in six patients associated with empty sella turcica (S1), hypoplastic adenohypophysis (S2, S5), or pituitary hypoplasia (S3, S4, S10). None had extra-pituitary cerebral anomalies on MRI (Table 1).

### 3.2. ProP1 Mutations

**Figure 2:**
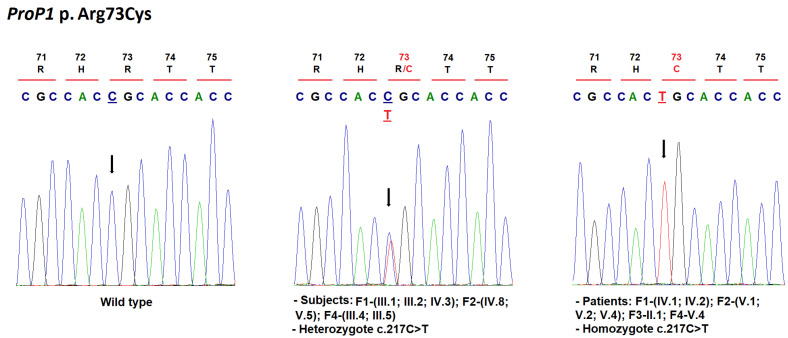
Mutation analysis of the *ProP1* gene in familial cases. Electropherogram analysis of *ProP1* gene in affected and unaffected individuals. The c.217C>T mutation in exon 2 resulted in substitution of the arginine by cysteine at amino acid position 73. Mutated nucleotide on the chromatographs is in bold, underlined and depicted with an arrow. Amino acid substitution is indicated in red.

**Figure 3:**
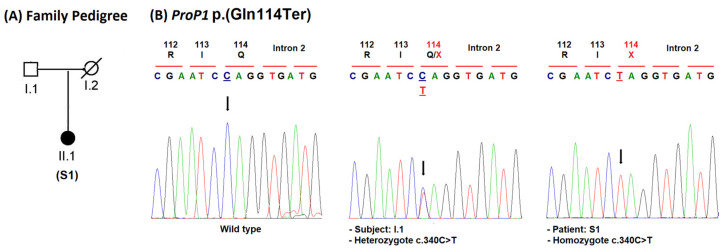
Mutation analysis of the *ProP1* gene in sporadic case. (**A**) Pedigree of the S1 sporadic case. (**B**) Electropherogram analysis of *ProP1* gene in the family. The c.340C>T mutation in exon 2 resulted in truncated protein lacking the last 112 codons [p.(Gln114Ter)]. Mutated nucleotide on the chromatographs is in bold, underlined and depicted with an arrow. Amino acid substitution is indicated in red.

**Figure 4:**
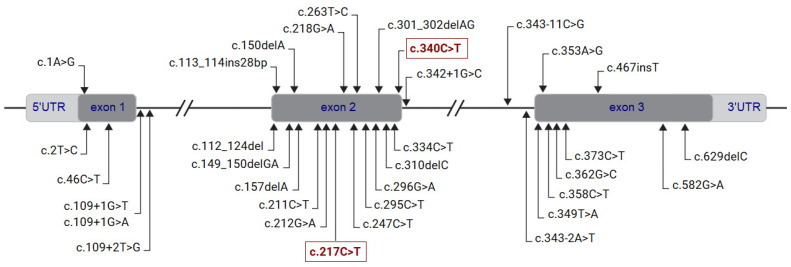
Location of *ProP1* mutations reported so far. Mutations found in our cohort are highlighted. Exons are represented by shaded boxes. Exons, introns, and untranslated regions (5′ and 3′UTRs) are not drawn to scale.

Overall, we identified eight patients (46%) carrying homozygous mutations in the *ProP1* gene (Table 1; Figure 2 and Figure 3). These mutations are located in exon 2 of the gene (Figure 4).

The previously reported mutation; p.Arg73Cys (c.217C>T; rs121917843) was detected in seven familial patients of our sample (Table 1, Figure 2). Patients’ parents and siblings with available DNA were heterozygous for the same mutation (Figure 2). The c.217C>T variant was found in the heterozygous state in two of 141,225 sequenced individuals in control populations from the GnomAD database (minor allele frequency (MAF): 7.08 × 10^−6^) and in one Tunisian control (MAF: 2.3 × 10^−3^). This variant was predicted to be deleterious by multiple in silico analysis tools. Reynaud et al. [30] investigated this mutation in vitro and showed a decreased affinity of the mutant protein to specific DNA binding sites, therefore preventing the activation of target genes.

Our patients with p.Arg73Cys mutations presented different combinations of hormone deficiencies (Table 1). All had GH deficiency at first diagnosis. They reached the age of puberty and showed clinical and hormonal findings consistent with hypogonadotropic hypogonadism upon admission or during the follow-up (Table 1). The corticotroph and thyreotroph axes remained functional in three patients at the last follow-up (Table 1). Two of these patients showed normal PRL hormone release (Table 1). MRI revealed normal pituitary gland size and morphology, adenohypophysis/pituitary hypoplasia with normal neurohypophysis, and empty sella turcica (Table 1).

We also found a novel homozygous transition in exon 2 (c.340C>T), which resulted in early termination of the protein at codon 114 [p.(Gln114Ter)] in a 10.3-year-old girl (Figure 3). At first examination, the S1 sporadic patient was diagnosed with GH, TSH, and ACTH deficiency (Table 1). Her cerebral MRI showed pituitary stalk interruption and empty sella turcica (Table 1). Since that time, she has no longer been coming to her doctor for an endocrine evaluation and the gonadotropin and PRL deficiencies were not diagnosed until she was 21 years old (Table 1). Her father was heterozygous for the mutation (Figure 3B). The p.(Gln114Ter) variant was absent in control populations from Tunisia (211 subjects) and from GnomAD. This new rare variant probably results in a complete loss of ProP1 function, as the resulting truncated protein lacks part of the homeobox and the entire transactivation domains [31].

We also confirmed by PCR the absence of large exon deletion or insertion in the remaining sporadic patients (S2–S8; Table 1).

## 4. Discussion

Our study was designed to expand the mutational spectrum of congenital non-syndromic CPHD. A cohort of 12 index patients and their relatives with various hormonal and pituitary phenotypes were screened for mutations in the *ProP1* gene.

We found the *ProP1* p.Arg73Cys mutation in all consanguineous families recruited (Figure 2 and Figure 4). This mutation was previously identified in three unrelated consanguineous Tunisian families [30,32], in two consanguineous Moroccan families [33], and in one patient of unknown ethnicity [34]. Only one French patient was a compound heterozygote for the p.Arg73Cys and p.Arg99Ter mutations [32]. Although the reason for the high frequency of the p.Arg73Cys mutation in our population remains unknown, a founder effect is, however, suspected to be responsible for the occurrence of CPHD in familial cases.

Consanguinity is common in North Africa and is estimated to account for ~50% and ~35% of all marriages in Tunisia and Morocco, respectively [35]. In their clinical epidemiologic study, Anwar et al. [35] emphasized the role of shared haplotypes, on which the founder mutation resides, in the occurrence of numerous recessive rare diseases in three Maghrebian populations (Tunisian, Algerian, and Moroccan). With immigration, many endemic disorders common in North Africa spread to many European countries decades ago, which is why some founder mutations were identified in these countries [35]. This may explain the occurrence of the p.Arg73Cys mutation in patients of French and unknown ethnicities.

In addition to the p.Arg73Cys mutation, two other frequent frameshift mutations (Figure 4) were analyzed for their geographic distribution and ethnic origin by Dusatkova et al. [7]. The first, c.301_302delAG, is widespread in patients from Eastern Europe, Latin America, and Portugal, and was introduced to these populations by two ancestral founders who originated from the Baltic Sea area 2525 years ago and from the Iberian Peninsula 583 years ago [7,36]. The second, c.150delA, is probably due to the ancestral founder haplotype introduced 1093 years ago in the Belarus region [7,37]. These common founder haplotypes were analyzed using 21 single-nucleotide polymorphism (SNP) markers flanking a 9.6-Mb region around the *ProP1* gene [7]. Another study reported two homozygous gross *ProP1* deletions (4.96 kb and 15.9 kb) in three Turkish families and one family from Northern Iraq [14]. In this study, haplotype analysis using a set of microsatellite and SNP markers revealed a shared haplotype of 350 kb (from rs4507507 to rs1110162, comprising the common D5S2008 marker) among *ProP1*-deletion carriers [14]. Additionally, two previous studies published in 1998 [38,39] analyzed the *ProP1* c.301_302delAG mutation and determined that it arose as an independent recurrent mutation rather than the result of a common ancestor.

Overall, the frequency of the *ProP1* mutation is higher in patients with familial than sporadic cases [6], which is in line with our findings. Only one additional *ProP1* mutation was found in the S1 sporadic patient (Table 1; Figure 3 and Figure 4). The previously unreported p.(Gln114Ter) nonsense variant was interpreted as pathogenic considering the concordance of hormonal and pituitary phenotypes (Table 1), the familial segregation analyses (Figure 3) and the predicted in silico deleterious effects on homeobox and transactivation domains [31]. Although several large cohorts of patients with mutant *ProP1* genes have been reported [6], a comparison of the clinical, hormonal, and imaging data for familial and sporadic cases revealed no genotype–phenotype correlation. The onset age and progressive loss of anterior pituitary hormonal deficiencies as well as pituitary imaging findings differ in *ProP1* patients with the same genotype, even in familial cases. Reported *ProP1* patients typically have clinical features of GH and TSH deficiencies in early childhood. They are usually diagnosed with delayed puberty due to gonadotropin deficiency. PRL is usually deficient. ACTH deficiency may progressively evolve at any time during the follow-up in childhood or adulthood [6]. In our cohort, *ProP1* patients had GH deficiency at the first examination and FSH/LH deficiency at the initial evaluation of delayed puberty (Table 1). Complete and partial ACTH deficiencies were observed during the first examination and at the follow-up (Table 1). TSH deficiency was not observed in two patients during the last follow-up (Table 1). Therefore, patients with normal thyreotroph and corticotroph function require permanent surveillance. Our patient cohort without *ProP1* mutation presented with complete or partial GH deficiency, TSH deficiency (except for S6 and S11 patients), FSH/LH deficiency (except for S5 and S10 patients), and ACTH deficiency (except for the S3 patient). PRL levels were normal in the majority of the cases (Table 1). Response to hormone replacement therapy was satisfactory in all patients and was in agreement with previous reports [6].

Overall, patients who reported with *ProP1* mutations lack extra-pituitary abnormalities [6], which is in line with our patients’ MRI results. Neuro-imaging of the pituitary region of reported *ProP1* patients usually shows normal or hypoplastic adenohypophysis, a topic neurohypophysis, and normal pituitary stalk. Occasionally pituitary hyperplasia evolving to hypoplasia and pituitary masses have also been observed [40,41,42]. Interrupted stalk and ectopic neurohypophysis are observed in the majority of reported CPHD patients [6]. Among our cohort of *ProP1* patients, two had empty sella turcica (F1-IV.2 and S1), one had pituitary stalk interruption syndrome (S1), and another had pituitary hypoplasia (F2-V.4). The pituitary phenotype of the remaining *ProP1* patients was in line with previously reported findings (Table 1).

## 5. Conclusions

We identified two *ProP1* mutations in five unrelated Tunisian families with non-syndromic CPHD. The most common mutation is the p.Arg73Cys. It was previously reported among several Maghrebian families with CPHD. Therefore, testing for this mutation in familial cases from the Maghrebian province presenting at least two anterior pituitary hormone deficiencies could ensure permanent surveillance and adequate genetic counseling. Additionally, our study demonstrates that the genetic etiology of non-syndromic CPHD has not been fully uncovered and involves novel genes that may unravel the complex mechanism of pituitary development and function.

## Figures and Tables

**Table 1:** Clinical features, hormonal follow-up and genotype of studied patients.

Case	Sex	Age at Diagnosis (Years)	Current Age (Years)	TSH (μU/mL)	FT4 (pmol/mL)	Basal LH (mU/mL)	Basal FSH (mU/mL)	Basal Testosterone (ng/mL)	Basal Estradiol (pg/mL)	GH (ng/mL)	Cortisol (ng/mL)	ACTH (pg/mL)	PRL (ng/mL)	MRI	*ProP1* Mutations
F1-IV.1	M	19.7	26	1.24	6.2 ^a^	0.1 ^a^	0.4 ^a^	0.3 ^a^	0.32 ^b^	Peak ^1^: 1.8 ^a^	Peak ^1^: 73 ^a^	11	2.5 ^a^	Normal	p.Arg73Cys
F1-IV.2	F	12.6	21.3	2.3	8.8 ^b^	0.42 ^a^	0.8 ^a^	0.01 ^b^	8 ^a^	Peak ^2^: 1.24 ^a^	Peak ^1^: 71 ^a^	10.5	6.74 ^b^	EST	
F2-V.1	M	8.6	14.2	1.1	7.4 ^a^	1 ^b^	1 ^b^	0.36 ^b^	0.12 ^b^	Peak ^2^: 0.23 ^a^	Peak ^1^: 37.5 ^a^	16	0.53 ^b^	APH, NPL	
F2-V.2	M	6.3	13.5	0.3	9.9 ^a^	0.2 ^b^	0.9 ^b^	0.06 ^b^	0.09 ^b^	Peak ^2^: 0.1 ^a^	Peak ^1^: 34.5 ^a^	15	1.1 ^b^	APH, NPL	
F2-V.4	F	13	23	1.13	12.2 ^b^	0.8 ^a^	0.9 ^a^	0.02 ^b^	9 ^a^	Peak ^1^: 0.04 ^a^	Peak ^1^: 61 ^a^	20	3 ^a^	PH	
F3-II.1	F	21.10	27	2.4	9.3 ^a^	0.7 ^a^	0.28 ^a^	0.01 ^b^	9 ^a^	Peak ^1^: 0.04 ^a^	Peak ^1^: 156 ^b^	17.2	1.71 ^a^	APH, NPL	
F4-V.4	M	7	16.8	2.2	12.7 ^b^	0.77 ^b^	1.5 ^b^	0.13 ^b^	0.4 ^b^	Peak ^2^: 1.4 ^a^	Peak ^1^: 53 ^a^	20.4	9 ^b^	Normal	
S1	F	10.3	21.5	1.1	3.15 ^a^	0.9 ^b^	1 ^b^	0.03 ^b^	8 ^b^	Peak ^1^: 0.5 ^a^	SO test: 53–40 ^a^	17	4.05 ^b^	PSIS, EST	p.(Gln114Ter)
S2	M	5	14	3.5	3.9 ^a^	0.1 ^b^	0.1 ^b^	0.3 ^b^	0.22 ^b^	Peak ^2^: 0.13 ^a^	SO test: 13.1–7.3 ^a^	11.6	2.5 ^b^	PSIS, APH, NPL	Absence of large exon deletion or insertion
S3	M	8	13	4.01	6 ^a^	1 ^b^	2.1 ^b^	0.01 ^b^	0.5 ^b^	Peak ^1^: 0.53 ^a^	Peak ^1^: 154 ^b^	17.3	13.5 ^b^	PSIS, PH	
S4	M	4	11	2.5	1.08 ^a^	0.1 ^b^	0.1 ^b^	0.1 ^b^	0.2 ^b^	Peak ^2^: 0.07 ^a^	Peak ^1^: 14.9 ^a^	20	0.5 ^b^	PSIS, PH	
S5	F	3.2	4.2	3.37	4.1 ^a^	Prepuberty period				Peak ^2^: 0.03 ^a^	Basal: 19.5 ^a^	26	11.47 ^b^	PSIS, APH, NPL	
S6	M	9.8	12	3.6	15.9 ^b^	0.19 ^a^	1.9 ^a^	0.8 ^a^	0.1 ^b^	Peak ^2^: 6.15 ^a^	Basal: 62.5 ^a^	15.3	6.86 ^b^	APH, NPL	
S10	M	5	6	3.3	5.2 ^a^	Prepuberty period				Peak ^2^: 1.5 ^a^	Basal: 67 ^a^	17	9.7 ^b^	PSIS, PH	
S11	M	14	20.2	1.14	14.8 ^b^	1.36 ^a^	0.9 ^a^	0.7 ^a^	0.2 ^b^	Peak ^2^: 0.36 ^a^	Peak ^1^: 85.5 ^a^	29	7.56 ^b^	PH	

Abbreviations: FT4: Free Thyroxine; TSH: Thyroid Stimulating Hormone; LH: Luteinizing Hormone; FSH: Follicle-Stimulating Hormone; GH: Growth Hormone; PRL: Prolactin; MRI: Magnetic Resonance Imaging; M: male; F: female; EST: Empty Sella Turcica; APH: Anterior Pituitary Hypoplasia; NPL: Normal Posterior Lobe; PSIS: Pituitary Stalk Interruption Syndrome; PH: Pituitary Hypoplasia; ^a^: anterior pituitary hormone deficiency diagnosed at first examination before starting replacement therapy; ^b^: patient developed anterior pituitary hormone deficiency during the follow-up; SO: investigation of the corticotropic axis by Synacthen test; ^1^: investigation of the somatotropic and the corticotropic axes by insulin-induced hypoglycaemia (IIH) test; ^2^: investigation of the somatotropic axis by glucagon-avlocardyl test. Normal reference ranges are mentioned in Section 2.1. Basal cortisol level was measured for all patients prior to performing the stimulation tests [IIH (30 min post-stimulation corresponding to blood glucose nadir) or Synacthen (1 μg, at 30 min and 1 h post-stimulation) tests]. Cortisol peak > 180 ng/mL indicates normal corticotroph function. A complete somatotropin deficiency was diagnosed when GH peak was less than 5 ng/mL in response to stimulation tests.

## Data Availability

The datasets analyzed in the present study are available from the corresponding author on reasonable request.
